# Effect of tibial tray backside design on stress transfer and micromotion in uncemented posterior‐stabilized TKA: A finite element study

**DOI:** 10.1002/jeo2.70608

**Published:** 2026-01-09

**Authors:** Zhenxian Chen, Zhangwen Ma, Bo Liang, Jianian Han, Jing Zhang, Yinghu Peng, Zhongmin Jin

**Affiliations:** ^1^ School of Construction Machinery Chang' an University Xi'an Shaanxi China; ^2^ Department of Bone Surgery Affiliated Hospital of Yan’ an University Yan'an Shaanxi China; ^3^ CAS Key Laboratory of Human‐Machine Intelligence‐Synergy Systems Shenzhen Institutes of Advanced Technology Chinese Academy of Sciences Shenzhen China; ^4^ Institute of Medical and Biological Engineering, School of Mechanical Engineering University of Leeds Leeds UK; ^5^ Tribology Research Institute, School of Mechanical Engineering Southwest Jiaotong University Chengdu Sichuan China

**Keywords:** interface micromotion, posterior‐stabilized prosthesis, stress transfer, tibial tray backside design, total knee arthroplasty

## Abstract

**Purpose:**

The uncemented posterior‐stabilized total knee arthroplasty (PS‐TKA) gained increasing acceptance as younger and active patients have higher requirements for the lifespan and functionality of prostheses, because their frequent gait and deep‐flexion activities may amplify tibial implant–bone interface micromotion and increase the risk of early tibial loosening. The purpose of this study is to evaluate the biomechanical fixation effects of three different tibial tray backside designs, with the aim of informing surgeons in selecting the most appropriate fixation strategy for uncemented PS‐TKA.

**Methods:**

The finite element and micromotion models were developed to quantify the tibial von Mises stress and interface micromotion under the knee loading conditions. The effects of three common tibial tray backside designs, including a cylindrical stem with a triple flat keel (CS‐TFK), a tapered stem with a double flat keel (TS‐DFK) and a cylindrical stem with a double serrated keel (CS‐DSK), on stress transfer and interface micromotion were compared.

**Results:**

During walking, all designs exhibited similar stress and micromotion patterns, with minimal risk for prostheses instability. During squatting, the cylindrical stem with double serrated keel design exhibited the highest proximal tibial stress (124.03 MPa) and the lowest maximum micromotion (361.91 µm), resulting in the largest area suitable for bone ingrowth (42.53%). In contrast, the tapered stem with double flat keel design had the lowest stress (90.70 MPa), and its area at risk for poor osseointegration (micromotion >150 µm) increased by 44.70% compared with the cylindrical stem with double serrates keel design.

**Discussion:**

The tibial tray backside design influenced its primary fixation. Among the evaluated designs, the CS‐DSK balanced stress transfer and interface micromotion, suggesting it may be favored for uncemented PS‐TKA in younger, active patients to lower the likelihood of early tibial aseptic loosening. CS‐DSK favored early ingrowth but produced higher local stresses, so overload risk should be monitored in older patients with osteoporosis.

**Level of Evidence:**

N/A.

**Abbreviations**
3D: three‐dimensional
CoCrMo: cobalt‐chromium‐molybdenum
CT: computed tomography
FE: finite element
FEA: finite element analysis
KOA: knee osteoarthritis
PCSA: physiological cross‐sectional area
PS: posterior‐stabilized
TKA: total knee arthroplasty
UHMWPE: ultra‐high molecular weight polyethylene

## INTRODUCTION

Knee osteoarthritis (KOA) is one of the most prevalent types of osteoarthritis, affecting approximately 16% of the global population [13]. Total knee arthroplasty (TKA) is widely recognized as the definitive intervention for end‐stage KOA, effectively alleviating pain, restoring joint function and enhancing patient quality of life [6]. Posterior‐stabilized (PS) knee prostheses, featuring a tibial post‐cam mechanism, are frequently employed to accommodate posterior cruciate ligament deficiency, but the high shear and compressive forces transmitted to the tibial component significantly increase the risk of aseptic loosening [31]. The cemented fixation uses the bone cement to bond the prosthesis to the bone [12], but potential limitations include fatigue, debonding at the cement–bone interface under high cyclic loads [41], bone cement implantation syndrome [18] and revision difficulty associated with cement removal [28]. In contrast, uncemented fixation relies on biologic osseointegration across the prosthetic backside and offers greater long‐term biological fixation due to enhanced osseointegration potential [5], but early interface micromotion is a critical determinant of success [14]. Nevertheless, aseptic loosening remains a critical clinical challenge and accounts for over 39% of TKA revisions, with tibial components demonstrating nearly twice the loosening rate of femoral components [23, 38, 40]. Consequently, optimizing the backside design of uncemented PS tibial trays has become an important research focus to reduce postoperative revision rates [2, 24, 32].

The backside structure of the tibial tray in PS‐TKA prostheses incorporates the essential design characteristics, such as stem, keel and column, which provide fixation and rotational constraint. Meneghini et al. [30] demonstrated that a keeled tibial tray achieved greater initial fixation stability than a double‐column design in normal bone models. Subsequently, David et al. [17] reported that keels with broader peripheral support transmitted torsional loads more effectively, thereby enhancing the mechanical performance of the tibial tray. More recently, Chong et al. [11] used micromotion models to show that stems with miniature keels significantly reduced bone–prosthesis interface micromotion compared with conventional stem‐only designs. Collectively, these studies suggest that the backside geometry of tibial trays plays a critical role in determining fixation mechanics and interface stability.

Tibial tray designs currently used in clinical practice are variable, including common configurations such as a cylindrical stem with triple flat keels (CS‐TFK), a tapered stem with double flat keel (TS‐DFK) [29] and a cylindrical stem with double serrated keel (CS‐DSK) [3]. Nevertheless, the effects of these tibial tray backside designs on proximal‐tibial stress transfer and bone–prosthesis fixation interface micromotion have rarely been systematically compared. Moreover, due to the post‐cam mechanism of PS prostheses, tibial postloading occurs especially during high knee flexion activities. Post‐cam engagement not only influences knee joint contact mechanics and kinematics [10] but also alters stress distribution and micromotion at the tibial fixation interface in PS‐TKA [20]. Therefore, clarifying how different backside geometries affect stress transfer and interface micromotion under postloading conditions is essential for selecting or designing tibial trays that minimize the risk of aseptic loosening in PS‐TKA.

In this study, we built a finite element model and a micromotion prediction model of PS‐TKA to quantitatively assess the effects of three common tibial tray backside geometries on stress transfer and interface micromotion during walking and squatting activities. We hypothesized that tibial tray backside geometry would significantly affect its primary fixation, and CS‐DSK would exhibit lower micromotion and a larger ingrowth‐favorable area than the other designs. This evaluation provides a mechanistic basis to guide the design and selection of uncemented PS tibial trays.

## MATERIALS AND METHODS

### Geometric models

A three‐dimensional (3D) geometric model of the left tibia was constructed based on open‐source computed tomography (CT) data (https://simtk.org/home/kneeloads) of a male patient (height: 180 cm, weight: 75 kg) using Mimics software (version 21.0, Materialise). The model was smoothed and encapsulated in Geomagic Studio (version 12.0, Geomagic), and subsequently converted into a 3D solid model in SolidWorks (version 2020, Dassault Systèmes).

**Figure 1:**
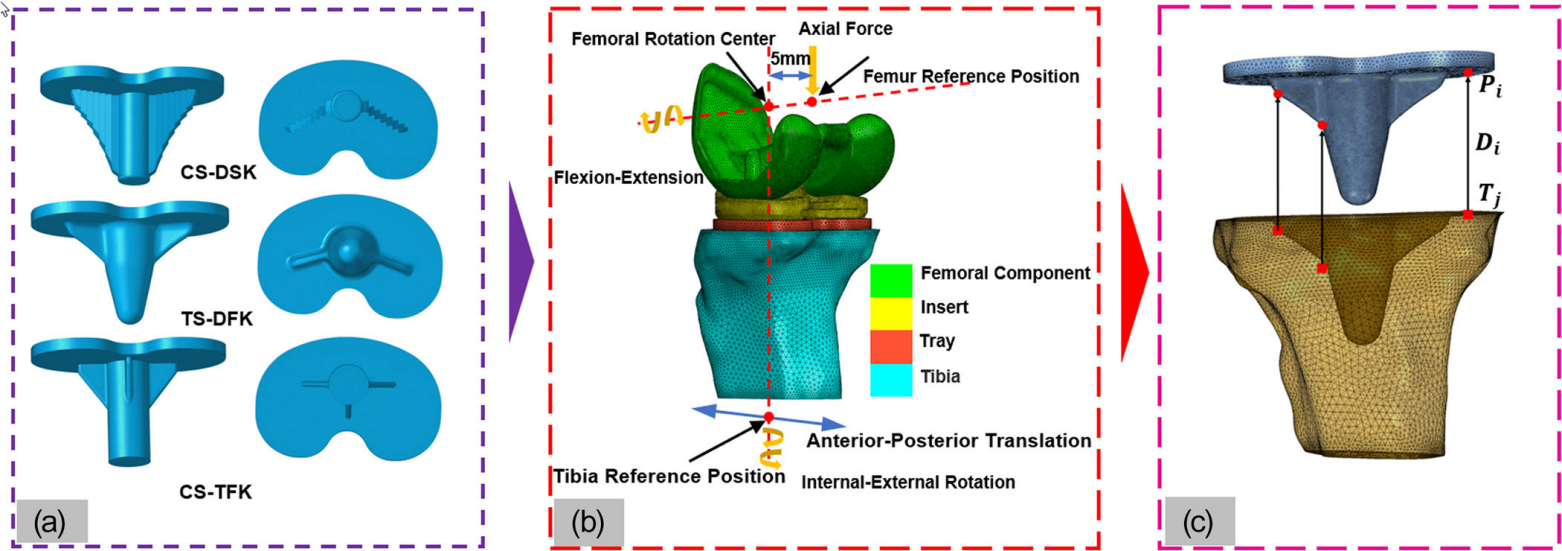
Workflow for geometric modeling, finite element analysis and micromotion calculation. (a) Geometric representations of the three tibial tray backside designs; (b) finite element model illustrating component segmentation, reference points, and applied boundary conditions; (c) node‐pairing scheme employed for micromotion prediction at the bone–prosthesis interface.

3D models of three commonly used PS‐TKA tibial trays, the PFC Sigma (Depuy), Attune (Depuy) and Scorpio NGR (Stryker), were established by performing inverse modeling in Geomagic Studio based on prosthesis point cloud data scanned with a 3D scanner (Figure 1a). These tibial trays differ in fixation design: PFC Sigma features a cylindrical stem with a triple flat keel (CS‐TFK), Attune features a tapered stem with a double flat keel (TS‐DFK), and Scorpio NGR features a cylindrical stem with a double serrated keel (CS‐DSK).

To isolate the effect of backside fixation geometry, the same femoral component and tibial insert (from the Attune prosthesis) were uniformly applied to all three tibial tray models. Virtual tibial osteotomy was performed in SolidWorks, followed by assembly of the tibial and femoral components to construct the complete 3D PS‐TKA model. The osteotomy plane was located 8 mm below the lateral tibial plateau, perpendicular to the mechanical axis in the coronal plane, with 0° posterior slope in the sagittal plane [46, 48].

### Finite element (FE) models

Three FE models of PS TKA with different tibial tray backside designs were established based on our previously validated FE modeling approach for PS‐TKA [20]. The 3D solid model of the PS‐TKA was imported into Hypermesh software (version 2020, Altair) for meshing bone and prosthesis. Based on the considerations of geometric model complexity, computational accuracy, and computational cost, all components were meshed with linear tetrahedral elements [34, 46]. Based on a mesh sensitivity analysis comparing 0.5, 1 and 2 mm element sizes, a global 2 mm was selected as the tibial mesh size because the relative errors were below 5%, with the contact surface refined to 0.5 mm. Meanwhile, the global size of the femoral component and tibial insert mesh elements was approximately 1.5 mm, with the contact surface refined to 0.7 mm. The tibial tray mesh elements were approximately 1.5 mm. Mesh quality was verified and optimized, with all elements exhibiting a Jacobian ratio between 0.8 and 1.0, and a length‐to‐width ratio below 5.0 [37].

The tibia was modeled as a nonhomogeneous, elastic and isotropic material, with material properties assigned based on the CT image gray value assignment method. Anisotropy was not considered. Bone density was distributed in the range of 0.02–1.45 g/cm^3^. After obtaining the bone density values, the elastic modulus of the tibia was determined using a previously established relationship between bone density and elastic modulus [1]. The femoral component was cobalt‐chromium‐molybdenum (CoCrMo) alloy with a modulus of elasticity of 210,000 MPa and a Poisson's ratio of 0.3 [45]. The tibial tray was titanium alloy (Ti6Al4V), with a modulus of elasticity of 105,000 MPa and a Poisson's ratio of 0.27; [45] and the tibial insert was nonlinearly elastic‐plastic ultrahigh molecular weight polyethylene (UHMWPE) with a modulus of elasticity of 463 MPa and a Poisson's ratio of 0.46 [47], and its plastic behavior was defined using experimentally derived true stress–strain data [19].

Penalty contact was defined between the tibial tray backside and tibia with a friction coefficient of 0.8, representing a roughened press‐fit bone–implant interface with high sliding resistance and consistent with experimentally informed modelling choices [39]. Similarly, a penalty contact was applied between the metal femoral component and the UHMWPE tibial insert with a friction coefficient of 0.04, as used in published studies [1].

Given the substantially higher elastic modulus of the femoral component compared with the tibial insert, the femoral component was modeled as a rigid body [9]. The tibial insert and tibial tray were constrained [37]. The flexion–extension axis was defined as the line connecting the rotation center of the femur and the femoral reference point. The femoral reference point was established by offsetting the femoral rotation center 5 mm medially along the flexion–extension axis. The tibial reference point was established by projecting the rotation center of the femoral component along the vertical direction [1].

The boundary conditions for joint forces and motions were obtained from previous musculoskeletal multibody dynamic simulations of PS‐TKA during walking and squatting simulations [10]. Walking represents a frequent, moderate, cyclic activity, whereas squatting represents a deep‐flexion activity. These activities dominate everyday use in younger, active recipients and provide a concise yet informative coverage of typical and high‐load physical conditions. The maximum total knee contact force was 1942.03 N during walking and 2296.53 N during squatting. During walking, the flexion–extension angle ranged from 5.49° to 60.02°, internal–external rotation from −0.63° to 1.25°, and anterior–posterior translation from −2.19 mm to 2.62 mm. During squatting, the corresponding ranges were 9.57° to 91.19°, −2.77° to 2.11° and −2.55 mm to 1.86 mm, respectively. Detailed values of knee joint forces and kinematics are provided in Supporting Information S1: Appendix A.

The total knee contact force and flexion–extension angle were applied to the femoral reference point. The varus–valgus rotation and superior–inferior translation of the femoral component were left unconstrained, while the remaining degrees of freedom were restricted. The anterior–posterior translation and internal–external rotation were applied to the tibial reference point, and the remaining degrees of freedom of tibia were constrained [34] (Figure 1b). The von Mises stress in the bone, as well as the relative displacement at the bone–prosthesis interface, were predicted using FE models.

### Micromotion prediction models

A micromotion prediction method, developed and validated by Yang et al. [46], was employed to construct the bone–prosthesis interface micromotion model for PS‐TKA, based on nodal displacement data. The displacements of element nodes located at the fixation interface of tibia and tibial tray backside were extracted from FEA results using custom Python scripts. For each node on the tibial tray backside, its nearest node on the opposing tibia was identified, and the distance between the two nodes was calculated before and after loading (Figure 1c). The change in this distance was defined as the local micromotion.

A region was classified as favorable for bone ingrowth when the micromotion was less than 50 µm, a threshold widely recognized to support reliable osseointegration [21, 33]. In contrast, regions exhibiting micromotion greater than 150 μm were considered at risk of fibrous tissue formation and subsequent long‐term loosening of the prosthesis [22, 44]. These thresholds were used to quantify and compare the fixation stability of the three tibial tray designs under walking and squatting.

Therefore, we divided the predicted micromotion values from the model into three regions: micromotion less than 50 µm, micromotion ranging from 50 to 150 µm and micromotion exceeding 150 µm. For each model, the coverage ratio of each region was calculated to provide a more comprehensive view of the fixation stability.

## RESULTS

### Von Mises stress of proximal tibia

**Figure 2:**
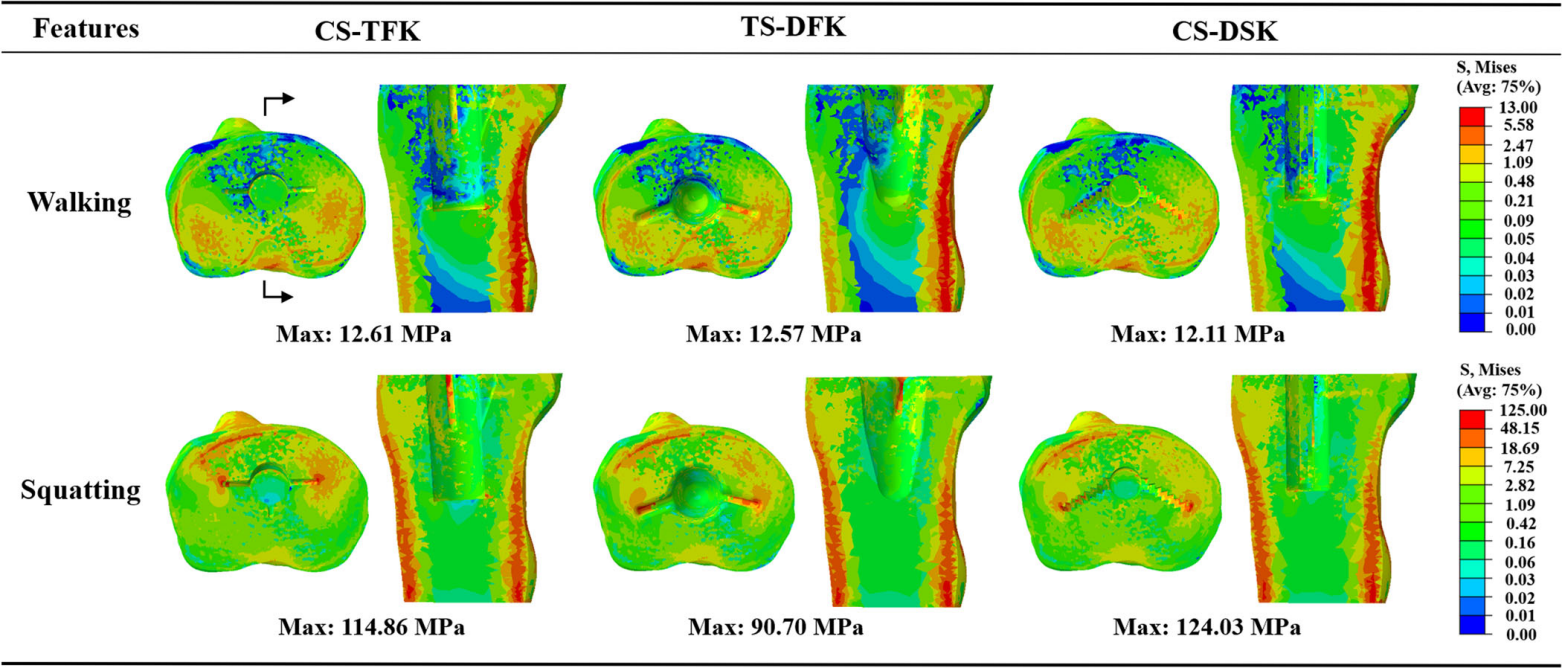
Von Mises stress distributions in the proximal tibia for the three tibial tray backside designs under walking and squatting activities.

Figure 2 shows the von Mises stress distributions in the proximal tibia for the three tibial tray backside designs under walking and squatting activities. During walking, the maximum von Mises stress values were 12.61, 12.57 and 12.11 MPa for the CS‐TFK, TS‐DFK and CS‐DSK, respectively. These stresses were confined to the posterior cortical bone. During squatting, the maximum stresses increased to 114.9, 90.7 and 124.0 MPa, with high‐stress regions observed along the anterior and posterior cortices and in areas adjacent to the keel. CS‐DSK showed the highest peak, with increases of 36.7% and 7.9% relative to TS‐DFK and CS‐TFK, respectively.

### Bone–prosthesis interface micromotion

**Figure 3:**
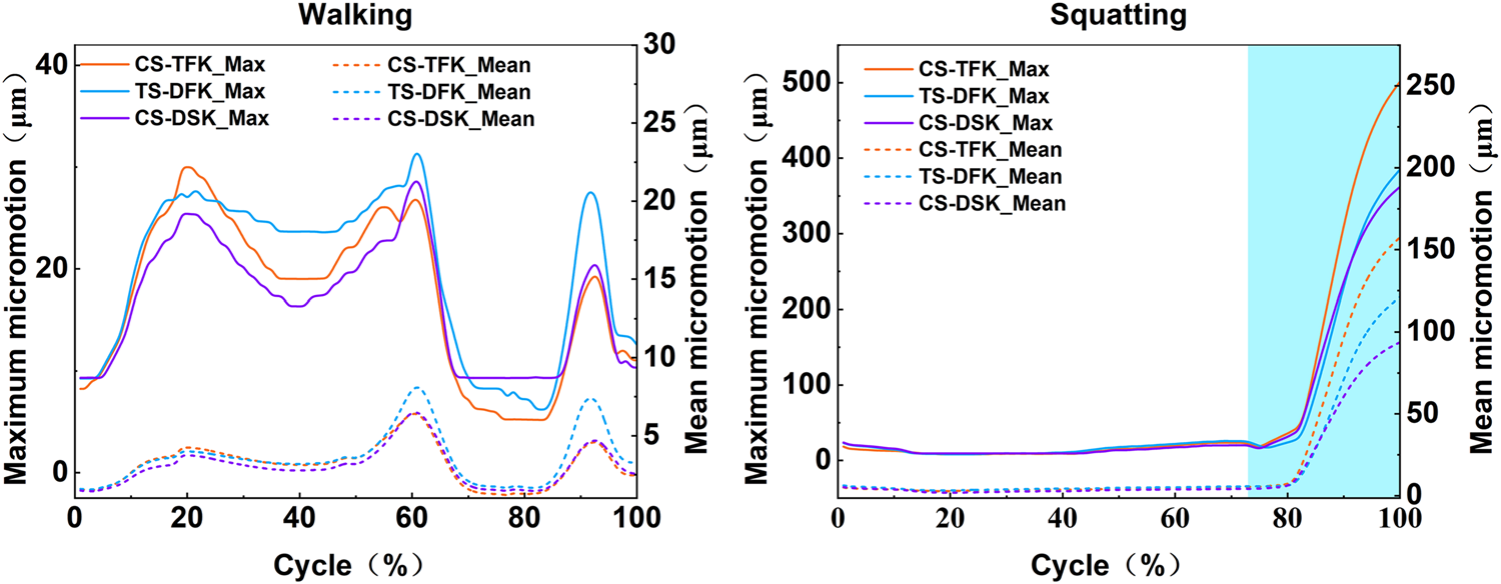
Variations in maximum and mean micromotion values for the three tibial tray backside designs under walking and squatting. The shaded areas in the figure indicate the intervals during activity due to the contact of the femoral component and the tibial post, resulting in post loading.

Figure 3 depicts the variations over the entire cycle in maximum (solid lines) and mean (dashed lines) interface micromotions for the CS‐TFK, TS‐DFK and CS‐DSK tray designs during walking and squatting. During walking, the maximum and mean micromotion curves of the three tray designs closely followed the joint‐force waveform. The TS‐DFK tray developed the largest excursion, with a maximum micromotion of approximately 32 µm, while its mean value remained below 9 µm. The CS‐TFK and CS‐DSK trays reached slightly lower maximum micromotion values of approximately 28 µm, and their mean micromotion likewise remained below 7 µm. During squatting, maximum micromotion stayed below 25 µm for all trays until the postcam began to engage at roughly three‐quarters of the squatting cycle. Once engagement occurred, micromotion rose sharply: CS‐TFK reached a maximum of 501 µm, TS‐DFK 385 µm, and CS‐DSK 362 µm, with CS‐TFK and TS‐DFK exceeding CS‐DSK by about 38% and 6%, respectively. Mean micromotion showed the same ranking, remaining below 160 µm throughout the cycle.

**Figure 4:**
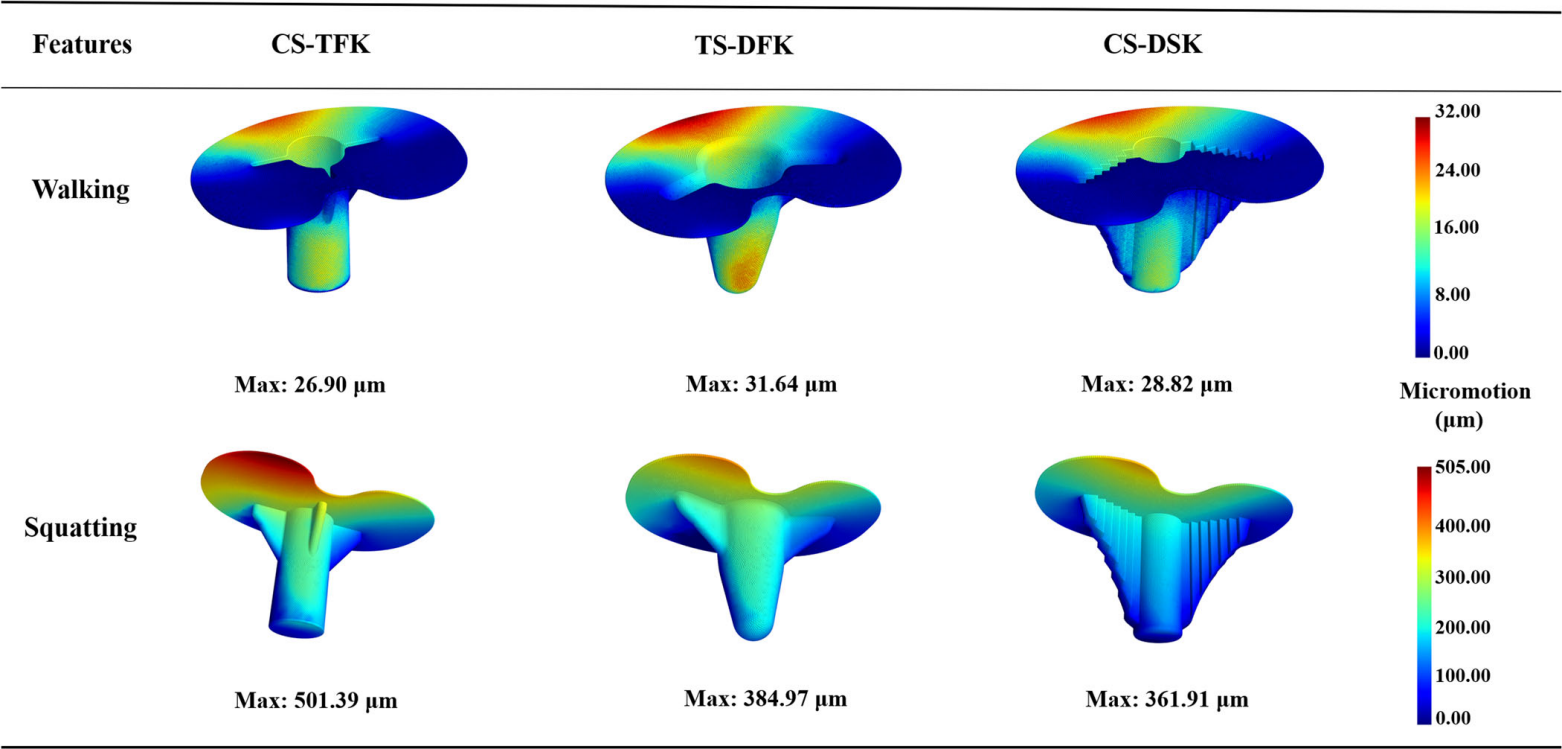
Micromotion distributions of the three tibial tray backside designs at 61% of the gait cycle during walking and at the maximum flexion angle during squatting.

Figure 4 illustrates the distributions of interface micromotion for the three backside designs at the maximum joint force moment (61% gait cycle) of walking and at the maximum flexion angle of squatting. During walking, elevated micromotion was confined to a narrow anterior rim in all trays; the TS‐DFK configuration showed the most concentrated hotspot of micromotion, whereas CS‐TFK and CS‐DSK exhibited slightly smaller and more uniform regions of micromotion. During squatting, high micromotion concentrated on the posterolateral rim of the tibial plateau for all designs. The CS‐TFK tray showed the broadest and most intense hotspot, extending from the posterolateral edge onto the posterior stem–keel junction. The TS‐DFK design exhibited a similar but smaller high‐micromotion patch. By contrast, the CS‐DSK tray restricted elevated micromotion to a narrow posterolateral rim of the tibial plateau, leaving the keel flanks and posterior stem largely unaffected.

### Magnitude distribution and threshold‐band areas

**Figure 5:**
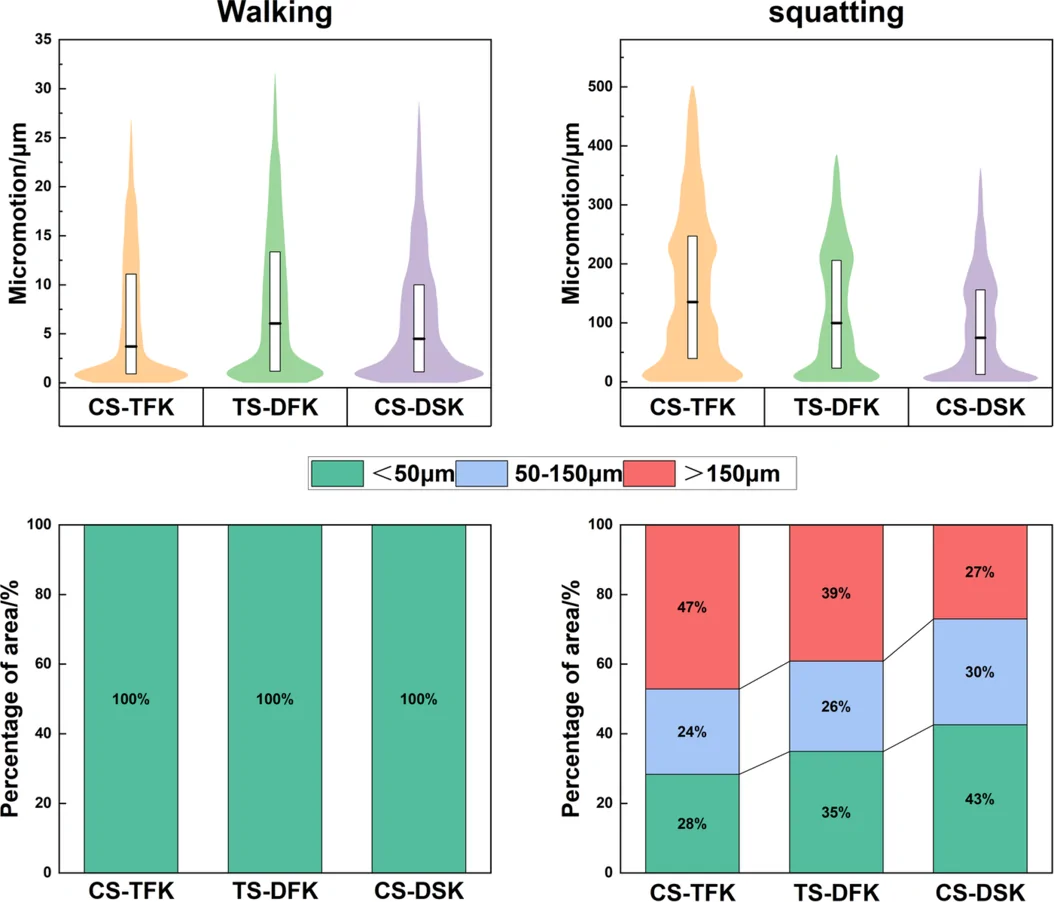
Distributions of micromotion magnitude and the percentage of interface area favorable for bone ingrowth at 61% of the gait cycle during walking and at the maximum flexion angle during squatting. The white box represents the interquartile range (25th–75th percentiles); the central line marks the median; the violin outline depicts the kernel‐density estimate. Colors denote clinically relevant thresholds: green ≤50 µm (favorable for bone ingrowth), blue 50–150 µm (limited ingrowth) and red >150 µm (risk of fibrous tissue and poor osseointegration).

Figure 5 summarizes both the magnitude distribution (upper violin plots) and the percentage of area favoring bone ingrowth (lower stacked bar charts) of micromotion for the three tibial tray designs at the maximum joint force moment (61% gait cycle) of walking and at the maximum flexion angle of squatting. During walking, the violin plots are slender and sharply tapered, indicating that virtually all nodes cluster at very low magnitudes; correspondingly, the stacked bar charts are exclusively filled with the region where micromotion is less than 50 µm. In squatting, the distributions broaden and extend upward, with marked differences among the designs. The CS‐TFK tray exhibits a pronounced upper tail, mirrored by the widest red segment, indicating that a substantial part of the interface experiences micromotion beyond 150 µm. The CS‐DSK tray shows a density concentrated toward the base of the violin and the largest green segment in the bar, reflecting the greatest proportion of micromotion below 50 µm and the smallest share above 150 µm. The TS‐DFK tray occupies an intermediate position, with its violin and bar partition lying between the other two designs. Overall, the area favoring bone ingrowth increased progressively from CS‐TFK to TS‐DFK to CS‐DSK, while the region associated with poor fixation shows the opposite trend.

## DISCUSSION

This study developed an FE model and a micromotion prediction model of PS‐TKA, and considered the postcam contact mechanism, which overcame the limitations associated with single activity modes and simplified mechanical assumptions prevalent in classic uncemented PS prosthesis studies. The biomechanical impacts of three tibial tray backside designs on the primary fixation stability of PS‐TKA prostheses were systematically analyzed. The von Mises stress in the proximal tibia and the micromotion at the bone–prosthesis interface were predicted, and the risk of aseptic loosening was evaluated.

The post‐cam contact of PS‐TKA prostheses can remarkably influence the stress transfer and interface micromotion [8, 20, 42], especially under high knee bend activities. In this study, the tibial von Mises stress was similarly distributed during walking, primarily located in the posterior cortical bone, consistent with the posterior load transfer characteristics of mid‐stance [43]. However, the postcam engagement produced a marked anteroposterior shear during squatting, together with the ongoing compressive load, generated an anteroposterior bending couple and lifted the posterior edge of the tray [25]. The tibial von Mises stresses were conspicuously increased, which localized in the anterior and posterior cortical bone rims and on both flanks of the keel, with the latter showing the most. The high micromotion zones shifted to the posterolateral region of the tibial tray. The postcam design of the Attune prosthesis results in delayed contact loading [10], which has a relatively minor impact. The postcam designs of PFC sigma and Scorpio NGR protheses led to a contact loading during entire walking and squatting [10], which may have a greater impact on bone stress transfer and interface micromotion.

Although the overall cortical bone stress distribution was comparable across the three designs, the peak tibial von Mises stresses were significantly influenced by the tibial tray backside geometry. Our findings underscore a practical two‐sided risk. On the one hand, the CS‐DSK design, characterized by double serrated keels, generated the highest tibial von Mises stress. Despite the advantage of extensive anchorage, local stress concentrations at the serration tips may elevate the risk of cortical microdamage or overload‐related trabecular fractures at the keel–cancellous bone interface [36]. On the other hand, the TS‐DFK design, featuring a tapered stem combined with smoother‐edged keels, exhibited the lowest tibial von Mises stress, suggesting improved load distribution and reduced local stress concentrations [4]. However, the two flat keels predominantly transferred load to cortical bone, potentially leaving peri‐stem cancellous bone under‐stimulated and prone to stress‐shielding‐induced adaptive resorption [16]. Thus, an optimal tibial tray backside design must carefully balance stress distribution, avoiding both stress shielding and cortical overload. Therefore, the CS‐DSK was recommended for patients with good metaphyseal bone quality, high activity levels and frequent deep‐flexion demands, which can minimize high‐risk micromotion (>150 µm) and support early osseointegration. TS‐DFK was recommended for osteopenic patients to obtain a lower peak proximal tibial cortical stress.

The tibial tray stem shape and keel geometry significantly influenced both the magnitude and distribution of interface micromotion, directly affecting osseointegration potential. During walking, all tray designs maintained micromotion below 50 µm, which is favorable for bone ingrowth [33]. However, significant differences emerged during high‐flexion squatting. The CS‐TFK tray displayed extensive micromotion regions exceeding 150 µm, indicating inadequate mechanical restraint by its triple flat keels under asymmetric loading conditions, leading to a higher risk of fibrous tissue formation [22]. Conversely, the CS‐DSK tray effectively confined elevated micromotion to a narrow posterolateral rim, maintaining the largest area within the optimal micromotion range and demonstrating superior conditions for osseointegration. The TS‐DFK design showed intermediate micromotion, more localized posteriorly. Thus, cylindrical stems combined with double serrated keels (CS‐DSK) provided the most favorable biomechanical environment for primary fixation stability, due to enhanced interlocking and more uniform pressure distribution [17, 26], followed by TS‐DFK, whereas CS‐TFK was associated with a higher risk of unstable fixation. These findings align with previous studies emphasizing the importance of anti‐rotational keel design features in enhancing implant stability under dynamic loading conditions [27, 35].

Comparative studies [5, 17, 26] of cementless tibial tray report that keel‐based, anti‐rotational geometries produce lower interface micromotion than central‐cone, no‐keel constructs under demanding loads. This supports the observation in this study that CS‐DSK most effectively curtails the >150‐µm high‐risk area. And recent meta‐analytic data [7, 15] indicate that modern cementless TKA achieves survivorship and aseptic‐loosening rates comparable to cemented fixation, particularly relevant for younger, active patients, the mechanistic trends (deep‐flexion sensitivity and the stabilizing role of anti‐rotational keels) are consistent with this study, which highlighted the clinical importance of early osseointegration via micromotion control.

This study has several limitations. First, only two physiological activities, walking and squatting, were considered. The effects of other common activities, such as running, stair ascent and descent, and sit‐to‐stand movements, were not evaluated. Different tibial tray backside designs may exhibit distinct biomechanical behaviors under various loading scenarios. Second, this study adopted a single biomechanical model, while the patient variability, including inter‐patient anatomical, bone mineral density, bone quality differences in elderly or osteoporotic patients, and gait patterns differences, and so on, was not considered in this study. These factors may change the absolute magnitudes of stress and interface micromotion. Third, this study evaluated only three widely used backside architectures. The keel, stem geometry (e.g., height, length, width, taper, spacing, or radius) design concepts (e.g., hybrid serrated–flat layouts, mini‐keels, or functionally graded porous Ti baseplates) as well as material variations should be considered in future studies. Fourth, the findings of this study were derived from computational simulations, which require further in vitro or clinical validation to strengthen it. Fifth, the micromotion thresholds and proximal tibial stresses quantify early mechanical conditions conducive or adverse to osseointegration, but they may not fully predict long‐term fixation. Future work should incorporate probabilistic, population‐level modeling and expand the activity set to high‐demand tasks such as stair descent, sit‐to‐stand, and kneeling to test the robustness of inter‐design rankings.

## CONCLUSION

The CS‐DSK design effectively balanced stress transfer and minimized interface micromotion, resulting in the largest area suitable for stable bone ingrowth and the smallest proportion of interface area experiencing high‐risk micromotion. However, its higher localized cortical stresses warrant caution and monitoring in patients with osteopenic, osteoporotic bone or thin cortices. These findings underscore the importance of backside geometry for uncemented PS‐TKA and can inform both implant design refinements and patient‐specific implant selection, particularly for younger, highly active patients.

## AUTHOR CONTRIBUTIONS

**Zhenxian Chen**: Conceptualization; project administration; writing—review & editing. **Zhangwen Ma**: Investigation; resources; writing original draft. **Bo Liang**: Software; visualization; writing original draft. **Jianian Han**: Software; formal analysis. **Jing Zhang**: Supervision; writing—review & editing. **Yinghu Peng**: resources; writing—review & editing; supervision. **Zhongmin Jin:** Methodology; supervision; writing—review & editing.

## CONFLICT OF INTEREST STATEMENT

The authors declare no conflicts of interest.

## ETHICS STATEMENT

This study did not involve experiments on human or animal subjects, and thus ethical approval was not required.

## Supporting information

Supporting information.

## Data Availability

The datasets generated and/or analyzed during the current study are available from the corresponding author on reasonable request.
