# Supplementary figures and images for: Effect of tibial tray backside design on stress transfer and micromotion in uncemented posterior‐stabilized TKA: A finite element study
Source: J Exp Orthop. 2026 Jan 9;13(1):e70608. doi: 10.1002/jeo2.70608 (PMC12784101; doi:10.1002/jeo2.70608)

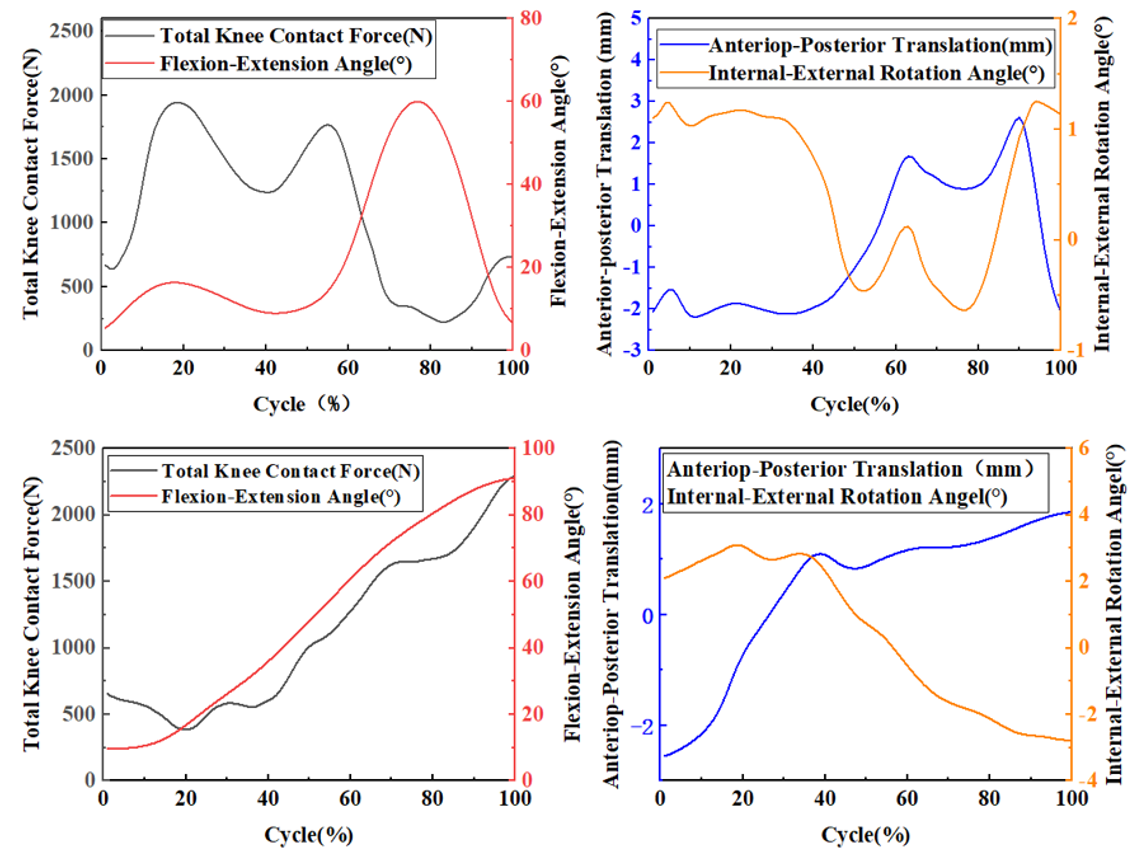

Supplement: Supplementary file 1 — Supporting information. [file JEO2-13-e70608-s001.tif]
